# Pathogenic variants carrier screening in New Brunswick: Acadians reveal high carrier frequency for multiple genetic disorders

**DOI:** 10.1186/s12920-022-01249-1

**Published:** 2022-04-29

**Authors:** Philippe Pierre Robichaud, Eric P. Allain, Sarah Belbraouet, Claude Bhérer, Jean Mamelona, Jason Harquail, Stéphanie Crapoulet, Nicolas Crapoulet, Mathieu Bélanger, Mouna Ben Amor

**Affiliations:** 1 Department of Medical Genetics, Vitalité Health Network, Dr. Georges-L.-Dumont University Hospital Centre, 330 University Ave., Moncton, NB E1C 2Z3 Canada; 2 Atlantic Cancer Research Institute, Moncton, NB Canada; 3 Department of Chemistry and Biochemistry, Université de Moncton, Moncton, NB Canada; 4 Centre de formation médicale du Nouveau-Brunswick, Moncton, NB Canada; 5 Department of Human Genetics, Faculty of Medicine and Health Sciences, McGill University, Montréal, QC Canada; 6 McGill Genome Center, Montréal, QC Canada; 7 Canada Excellence Research Chair in Genomic Medicine, McGill University, Montréal, QC Canada; 8 Research and Scientific Development, Vitalité Health Network, Moncton, NB Canada; 9 Department of Family and Emergency Medicine, Université de Sherbrooke, Sherbrooke, Canada; 10 Department of Pediatrics, Université de Sherbrooke, Sherbrooke, QC Canada

**Keywords:** Acadians, Carrier screening, Founder population, Inherited disease

## Abstract

**Background:**

Founder populations that have recently undergone important genetic bottlenecks such as French-Canadians and Ashkenazi Jews can harbor some pathogenic variants at a higher carrier rate than the general population, putting them at a higher risk for certain genetic diseases. In these populations, there can be considerable benefit to performing ethnic-based or expanded preconception carrier screening, which can help in the prevention or early diagnosis and management of some genetic diseases. Acadians are descendants of French immigrants who settled in the Atlantic Coast of Canada in the seventeenth century. Yet, the Acadian population has never been investigated for the prevalence/frequency of disease-causing genetic variants.

**Methods:**

An exome sequencing panel for 312 autosomal recessive and 30 X-linked diseases was designed and specimens from 60 healthy participants were sequenced to assess carrier frequency for the targeted diseases.

**Results:**

In this study, we show that a sample population of Acadians in South-East New Brunswick harbor variants for 28 autosomal recessive and 1 X-linked diseases, some of which are significantly more frequent in comparison to reference populations.

**Conclusion:**

Results from this pilot study suggests a need for further investigation of genomic variation in this population and possibly implementation of targeted carrier and neonatal screening programs.

**Supplementary Information:**

The online version contains supplementary material available at 10.1186/s12920-022-01249-1.

## Background

Preconception ethnicity-based carrier screening allows to assess the potential risk of inheritance for certain genetic disorders [1]. This screening approach can be particularly effective in founder population known to have a high prevalence of certain genetic disorders due to the founder effect. This practice has greatly benefited some ethnic groups such as Ashkenazi Jews [2, 3]. However, some ethnic populations presenting genetic bottlenecks are still underserved and could benefit from preconception genetic counseling.

Acadians are descendants of European migrants who settled in the Atlantic coast of Canada, mainly in Nova-Scotia and New Brunswick, during the French colonization of North America during the seventeenth century. The population is thought to originate from approximately 50 French families who settled before 1650 and whose descendants expanded to approximately 13,000 Acadians at the start of the Deportation [4–6]. Acadian exiles have left descendants across the Americas. Notably, Acadians have settled in Quebec and Louisiana, and thus share some degree of common ancestry with populations in Quebec and their “Cajun” relatives in the United-States [7–10]. Today, most of the Acadian population resides in Canada’s Atlantic provinces. Population genetics studies from subgroups of Acadians have demonstrated their lower genetic diversity, and genetic differentiation from other European-descent populations from North America. They are therefore thought to be genetically distinct from other North American populations due to their unique history.

This lower genetic diversity coupled with the identification of disease-causing variants in certain families likely places Acadians, at higher risk for certain autosomal recessive diseases [7–9, 11, 12]. Pathogenic genetic variants for Usher Syndrome, Friedreich Ataxia, Tay-Sachs syndrome and Fanconi syndrome have been described in some Acadian families [7, 9, 11–15]. Yet, a systematic assessment of the frequency of disease-causing variants in Acadians is still lacking. In order to establish an initial targeted screening program, an expanded carrier screening for the target population must be undertaken to narrow down the pool of potential pathogenic variants which may be relevant to this population. We therefore sought to perform an expanded screening of known frequent autosomal recessive and X-linked conditions in Acadians native to New Brunswick (NB), Canada. Given the scarcity of genetic information available for Acadian populations in NB, the overarching goal is to assess the usefulness of a standardized carrier screening program for couples within this population who wish to conceive.

The objectives of this pilot project were to determine possible founder variants and frequent genetic conditions in people native to NB, particularly, the South-East portion of NB were historically a large proportion of Acadian are living. This is the first genetic study of Acadians to demonstrate the relevance of conducting more in-depth studies across the province. Our results show a high frequency of certain variants in *AIRE*, *BCHE*, *ETFDH*, *RAPSN* and *SLC17A5* (2 variants), among others, within a sample population of 60 individuals from South East NB. The carrier frequency of several of these variants was higher than expected in our sample, and even much higher than other high-risk sub-populations for the same disease. This indicates that carrier screening in NB Acadians could be warranted for some regions. Subsequent studies may be needed to verify if these results are applicable to other Acadian or Native American communities and to further elucidate population structure in Atlantic Canada.

## Methods

### Recruitment

We recruited individuals 19 years or older living in the South-East NB and having at least one Acadian grandparent born in NB and a valid NB Medicare number were included in the study. Couples awaiting newborns were excluded to avoid undesirable outcomes after the 20-week limit on action. Participants were recruited at the Dr. Georges L.-Dumont University Hospital Centre and the Greater Moncton Family Medicine Unit. Participants were introduced to the research project by participating physicians and with information pamphlets. Details about the project were discussed with the patient prior to obtaining informed consent. Medical history and ancestry were gathered through a questionnaire. Participants carrying a pathogenic variant had an extensive review of their family history and were asked if any other known members of their families had participated in the study. This ensured that any 1st or 2nd degree relatives were not included within the 60 participants. The presence of 3nd degree relatives is possible. Our recruitment strategy therefore minimized the risk of any calculated allele frequencies being heavily impacted by relatedness.

### DNA sampling

DNA samples were collected using the Oracollect DNA buccal swab from DNA Genotek inc. (Ottawa, On, CA) provided by Fulgent Genetics (Temple City, CA, USA) who was contracted to perform the clinical carrier screening. The DNA from oral swabs was extracted by Fulgent Genetics using standard bead-based methods on an AnaPrep 12 system from BioChain (San Francisco Bay, CA, USA).

### Expanded carrier screening

A sequencing panel of 312 genes associated with autosomal recessive disorders, as well as 30 X-linked genes were included in the panel (Additional file 1: Table S1). These genes were chosen based on reported genetic diseases with increased risk in French-descent ethnic populations, such as Acadians, Cajuns and French-Canadians from Quebec [7, 9, 16]. Sequencing and data analysis of carrier screening data for inherited disease was performed by Fulgent Genetics (Temple City, CA, USA).

Next-generation sequencing libraries were generated using a modified version of the KAPA DNA Library Preparation Kit from Roche Sequencing (Pleasanton, CA, USA). Briefly, DNA was enzymatically fragmented, perform end repair and ligate adapters. The fragmented DNA was then amplified by standard PCR, which simultaneously added samples-specific barcodes. The coding exons of targeted genes were enriched using a hybrid of proprietary and commercial capture set probes from Integrated DNA Technologies inc. (Coralville, IA, USA). Prepared DNA libraries were then sequenced using a HiSeqX or NovaSeq 6000 from Illumina inc. (San Diego, CA, USA).

Sequence alignment and variant calling were performed using Sentieon’s germline variant calling pipeline (v2018.08.05) (Sentieon Inc., San Jose, California, USA) [17]. All genomic regions in this article use the GRCh37/hg19 human reference genome. Following alignment, variants were detected in regions of at least 10 × coverage. Coding regions and splicing junctions of genes listed had been sequenced with coverage of at least 10× and 20×, respectively, by NGS. The remaining regions did not have 10 × coverage and were not evaluated. Genetic variants were classified using technical standards established by the American College of Medical Genetics and Genomics (ACMG), Association for Molecular Pathology (AMP) and ClinGen [18–20]. Variants were interpreted using locus specific databases, literature searches, and other molecular biological principles. To minimize false positive results, any variants measured with a quality scores below 500 (roughly 40×) are confirmed by Sanger sequencing. Deletions and duplications were also investigated in all the genes included in the panel.

### Data analysis

We compared the allele frequencies observed in our Acadian sample to the general European populations sampled in the gnomAD database version 2. Specifically, for pathogenic variants detected in our sample, we extracted allele frequencies in gnomAD non-Finnish Europeans (NFE) computed in whole exome and whole genome data. Furthermore, we calculated heterozygous carrier frequencies and compared it to gnomAD Non-Finnish Europeans heterozygotes frequencies.

$$Heterozygote \,Frequencies = \frac{{\left( {Positive\, alleles - 2\left( {Homozygotes} \right)} \right)}}{{\left( {0.5 \times Total \,alleles} \right)}}$$

For each variant present in populations, we computed the allele frequency ratio in Acadians versus gnomAD NFE, as a measure of the relative frequency enrichment. To test for the difference in allele frequencies between Acadians and NFE, we used a Fisher-exact test (one-sided) on allele counts. We computed the relative risk of being a carrier as a ratio between observed frequencies on gnomAD frequencies. Comparative analyses of Acadians with other reference populations with high carrier frequencies for identified disease-linked variants were also performed, based on data from the literature and gnomAD.

Expected live births in NB were estimated using the most recent available census data from Statistics Canada and the Government of NB [21, 22]:

$$X = q^{2} \left( {B \times \frac{A}{N}} \right)$$

where *X* is the expected number of live births, *q*^*2*^ is the number of affected individuals with homozygous genotypes for a pathogenic variant, based on observed allele frequencies and Hardy–Weinberg equilibrium, *A* is the number of self-identified New Brunswickers of French or Acadian origins and *N* is the total population of NB. Allele frequency ratios (AF Ratios) were calculated by dividing the allele frequencies of Acadians by gnomAD allele frequencies for the same variant in Non-Finnish Europeans (NFE).

### Result communication

All participants with identified pathogenic variants and who consented to receiving their results had a genetic counselling session over the phone with a geneticist. Results were communicated, conditions were explained, personal and family history were reviewed. Medical recommendations were given when applicable. A family letter in their desired language as well as a copy of their molecular report were posted to each participant to share with at risk family members who desire a screening for their carrier status for the identified variants.

## Results

### Carrier screening reveals multiple pathogenic variants in Acadians

**Fig. 1:**
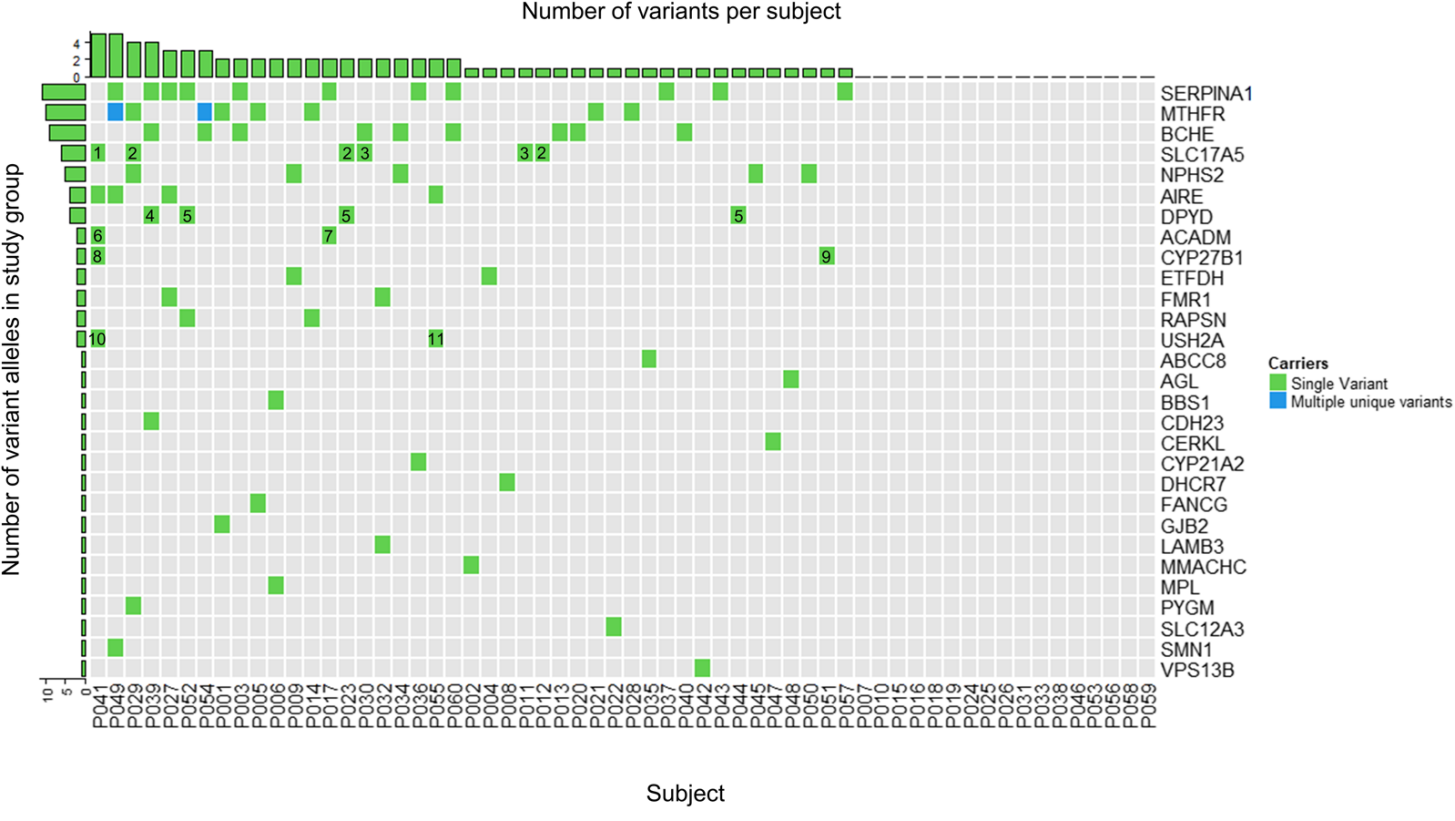
Distribution of the pathogenic variants in the 60 participants. The two variants were detected in the MTHFR (NM_005957.4) gene: the c.665C > T variant only (in green) or c.665C > T and c.1286A > C (in blue). Three variants were found in SLC17A5 (NM_012434.4): 1 c.533del, 2 c.918T > G and 3 c.819 + 1G > A. Two variants were found in DPYD (NM_000110.3): 4 c.1905 + 1G > A and 5 c.2846A > T. Two variants were present in ACADM (NM_000016.5): 6 c.199T > C, 7 c.250C > T. The CYP27B1 gene (NM_000785.3) also harbored two variants: 8 c.1319_1325dup, 9 c.262del. The USH2A gene (NM_206933.2) also had two variants: 10c.4338_4339del, 11 c.2276G > T. The participant P009 was homozygous for the NPHS2 variant and participants P005 and P025 were homozygous for the MTHFR variant

Sixty individuals from South-East NB consented to participate in this study. Participants characteristics are shown in Additional file 1: Table S2. The expanded carrier screening revealed 36 pathogenic variants in 29 genes (Fig. 1) involved in the etiology of 28 autosomal recessive disorders and one X-linked disorder (Additional file 1: Table S3). Overall, 43 of the 60 participants were carrier of at least one pathogenic variant in the expanded panel of 342 genes, corresponding to 71.7% of the sample. In addition, 21 participants (35%) carried more than one pathogenic variant, with up to five pathogenic variants observed in one participant. For *MTHFR, DPYD, ACADM, CYP27B1* and *USH2A*, two different pathogenic variants were observed. Three different pathogenic variants were found in *SLC17A5*. Furthermore, two individuals harbored two separate unique pathogenic variants for the *MTHFR* gene. However, it was impossible to determine if these participants were compound heterozygotes, as both variants were characterized by short-read sequencing technology and their genomic coordinates were relatively far apart (621 bp). Therefore, we could not conclude if those variants were on *cis* or *trans* alleles. Two participants were homozygous for the *MTHFR* (c.665A > C) and one participant was homozygous for the *NPHS2* variant. The majority of individuals with these *MTHFR* and *NPHS2* variants are healthy or have mild symptoms [23–26]. The most frequent variation was a pathogenic missense variant in *SERPINA1* (c.863A > T), which was detected in 11 individuals. More detailed individual variants frequencies are presented in Table 1.

**Table 1:** The observed allele frequency (AF) and heterozygous frequency (HF) compared to the non-Finnish Europeans from the gnomAD database

Genes	Variants	AF Acadian sample	AF gnomAD NFE	AF ratio (Acadian/NFE)	HF Acadian Sample	HF gnomAD NFE	HF Ratio (Acadian/NFE)	Adj. *p* values
*ABCC8*	c.2252_2253dup	0.00833	NA	NA	0.01667	NA	NA	NA
*ACADM*	c.199T > C	0.00833	0.00102	8.2	0.01667	0.00203	8.2	1.000
*ACADM*	c.250C > T	0.00833	0.00012	67.2	0.01667	0.00025	67.2	0.486
*AGL*	c.18_19del	0.00833	0.00009	89.7	0.01667	0.00019	89.7	0.372
*AIRE*	c.967_979del	0.03333	0.00096	34.6	0.06667	0.00193	34.6	< 0.001
*BBS1*	c.1169T > G	0.00833	0.00277	3.0	0.01667	0.00555	3.0	1.000
*BCHE*	c.293A > G	0.07500	0.01766	4.2	0.15000	0.03439	4.4	0.009
*CDH23*	c.5371G > T	0.00833	NA	NA	0.01667	NA	NA	NA
*CERKL*	c.847C > T	0.00833	0.00054	15.5	0.01667	0.00108	15.5	1.000
*CYP21A2*	c.293-13C > T	0.00833	NA	NA	0.01667	NA	NA	NA
*CYP27B1*	c.262del	0.00833	0.00009	97.3	0.01667	0.00017	97.3	0.369
*CYP27B1*	c.1319_1325dup	0.00833	0.00017	48.8	0.01667	0.00034	48.7	0.658
*DHCR7*	c.964NA1G > C	0.00833	0.00608	1.4	0.01667	0.01216	1.4	1.000
*DPYD*	c.1905 + 1G > A	0.00833	0.00566	1.5	0.01667	0.01130	1.5	1.000
*DPYD*	c.2846A > T	0.02500	0.00516	4.8	0.05000	0.01029	4.9	0.771
*ETFDH*	c.51dup	0.01667	0.00014	119.3	0.03333	0.00028	119.3	0.005
*FANCG*	c.1480 + 1G > C	0.00833	0.00004	189.6	0.01667	0.00009	189.6	0.195
*FMR1*	47 CGG repeats	0.01667	NA	NA	0.03333	NA	NA	NA
*GJB2*	c.35del	0.00833	0.00958	0.9	0.01667	0.01903	0.9	1.000
*LAMB3*	c.2842del	0.00833	0.00004	215.2	0.01667	0.00008	215.2	0.172
*MMACHC*	c.331C > T	0.00833	0.00009	89.3	0.01667	0.00019	89.3	0.374
*MPL*	c.305G > C	0.00833	0.00071	11.7	0.01667	0.00142	11.7	1.000
*MTHFR*	c.665C > T	0.08333	0.33800	0.2	0.10000	0.44324	0.2	1.000
*MTHFR*	c.1286A > C	0.01667	0.31730	0.1	0.03333	0.42937	0.1	1.000
*NPHS2*	c.686G > A	0.05000	0.03099	1.6	0.06667	0.06938	1.0	1.000
*PYGM*	c.148C > T	0.00833	0.00252	3.3	0.01667	0.00503	3.3	1.000
*RAPSN*	c.133G > A	0.01667	0.00007	238.9	0.03333	0.00014	238.9	0.001
*SERPINA1*	c.863A > T	0.09167	0.03668	2.5	0.18333	0.06973	2.6	0.149
*SLC12A3*	c.248G > A	0.00833	0.00003	268.4	0.01667	0.00006	268.4	0.144
*SLC17A5*	c.533del	0.00833	0.00010	82.8	0.01667	0.00020	82.7	0.401
*SLC17A5*	c.819 + 1G > A	0.01667	0.00001	1884.1	0.03333	0.00002	1884.0	< 0.001
*SLC17A5*	c.918T > G	0.02500	0.00004	645.7	0.05000	0.00008	645.6	< 0.001
*SMN1*	Absence of SMN1	0.00833	NA	NA	0.01667	NA	NA	NA
*USH2A*	c.2276G > T	0.00833	0.00142	5.9	0.01667	0.00283	5.9	1.000
*USH2A*	c.4338_4339del	0.00833	0.00001	944.2	0.01667	0.00002	944.2	0.066
*VPS13B*	c.6002del	0.00833	0.00002	358.4	0.01667	0.00005	358.4	0.115

Frequency ratio for each variant was calculated using the ratio of frequencies from the sample group and gnomAD Non-Finnish Europeans. Fisher’s one-tailed exact test adjusted using Bonferroni correction

### Pathogenic variants at higher frequency in Acadians compared to other European-descent populations

We compared our allele frequency observed in Acadians to NFE allele frequencies in gnomAD (Table 1). Allele frequency ratios (Acadians/gnomAD NFE) in the study population were higher than expected for 29 out of 36 pathogenic variants (Table 1). In total, six variants in five genes (*AIRE*, *BCHE*, *ETFDH*, *RAPSN*, and *SLC17A5*) were significantly enriched in the study group after Bonferroni correction (Table 1). Two pathogenic variants in *SLC17A5* gene causing sialic acid storage disorder were among the most enriched statistically significant variants in the study group compared to the reference population (Table 1). One variant in *USH2A*, while not statistically significant, did show a strong trend for enrichment (*P* < 0.1, Table 1). Afterwards, we compared Acadians to ethnic groups with known genetic enrichment in all observed variants, based on data from gnomAD (Additional file 1: Table S4). These comparisons indicated that Acadians still displayed significantly higher carrier frequencies when compared to reference populations with relatively high allele frequencies for these variants, suggesting increased risk for these diseases in Acadians. In addition, our data does not deviate from Hardy–Weinberg equilibrium for these variants (Additional file 1: Table S5).

We estimated the number of affected live birth per year for the five genetic diseases caused by the six statistically significant pathogenic variants identified in this study. We assumed Hardy Weinberg equilibrium and 192,775 self-identified Acadians from the 2016 Canadian Census [21] (see “Methods” section). We estimate 0.5 affected birth every year for glutaric aciduria IIC disease and congenital myasthenic syndrome, 1.5 per year for sialic acid storage disorder, 1.8 for autoimmune polyendocrinopathy syndrome type I and up to 9 per year for Butyrylcholinesterase deficiency if both parents are ethnic Acadians (Table 1).

### Variants identified by carrier screening are linked to some individual`s family history of disease

Although not conducted as a confirmatory procedure, some post-screening clinical consultations corroborated some of the genetic screenings performed. For example, one participant identified as a carrier for *USH2A* gene had a family history of retinitis pigmentosa in her offspring. Another participant was found to be a carrier for a variant in the *BCHE* gene and had a family history of prolonged apnea post anaesthesia in second degree relatives.

## Discussion

Preconception carrier screening has become an important tool for assessing the risk of inheriting genetic disorders for many ethnic populations. While some genetic studies have examined genetic variability in French-descent populations of Canada, such as French-Canadians from Québec and other related sub-populations, this is the first study to examine the frequency of disease-causing genetic variants in Acadians from NB. This surprising lack of genetic characterization has gone on in spite of their notably small founder population and discovery of disease-causing variants in families of Acadian ancestry distinct from diseases observed in populations from Quebec [7–10, 27].

Many countries with at-risk ethnic groups perform ethnic-based carrier screening to detect frequent pathogenic variants in their population. In this study, we have assessed genetic variation in 342 disease-causing genes and identified a higher prevalence of several pathogenic variants in a subset of the Acadian population compared to general European population. Our data suggests a higher risk for some autosomal recessive diseases in Acadians, notably those associated with *AIRE*, *BCHE*, *ETFDH*, *RAPSN* and *SLC17A5* (2 variants). As such, our results reveal the need for a permanent preconception screening program for couples of Acadian origins, whether it be in the form of a targeted assay or a broader expanded screening. Ethnicity-based panels have been implemented for some time, but as sequencing costs diminish over time, expanded carrier panels are increasingly being offered to couples wishing to conceive. This is especially relevant in ethnically and culturally diverse countries where populations are less homogeneous. This does present some advantages over traditional single-disease and ethnic-based screening, but largely depends on affordability, local healthcare priorities, demand and resources [28–30]. For Acadians, it will be of utmost importance to sample other regions within NB that are demographically majority Acadian to elucidate population structure and degree of homogeneity. If only a few recurring variants are found targeted screening may be preferential, while expanded screening may be warranted if the NB Acadian population demonstrates high heterogeneity and within-group stratification. In any case, it is important that an initial expanded carrier screening be done to establish the identity and risk of genetic disease in the Acadian population.

While our study has revealed interesting results in the Acadian population, it does have some limitations. Estimations of affected births are based on the presumption of relative genetic similarity between south-eastern NB and other Acadians in the province. However, this may not be the case and depends highly on the level of stratification in NB Acadian populations. Furthermore, the sequencing technologies and the approaches chosen for this study are unable to detect novel pathogenic variations in non-coding regions, some CNVs or inversions. Heritable epigenetic alterations may also be present. A more complete analysis of this population with whole-genome sequencing and other technologies may therefore reveal further molecular traits enriched in Acadians. This pilot study also had a relatively small sample size. Many pathogenic variants were only measured once in our study group, and despite the very low probability of measuring even one variant allele in a sample size of 60 (Additional file 2: Figure S1) we cannot conclude that these variants are enriched in Acadians. One specific *USH2A* variant identified in our study was consistent with previous literature in Acadian families, however other previously described variants in Acadians were not identified in our group [7, 9]. Future genetic studies in Acadians with larger sample sizes and more diverse geographical sampling may help in identifying truly enriched variants in this population.

It should be noted that although some genes measured in this study are interesting for investigating founder effects or general characterization of the genetic landscape for Acadians, some should not be considered for preconception carrier screening. For example, the *MTHFR* variants included in this study are considered ‘risk alleles’ rather than pathogenic and are found in the reference populations at a relatively high frequency [26]. In addition, variants in some genes, such as *SERPINA1* are associated with late-onset diseases that may not be required for preconception carrier screening but are nonetheless interesting for diagnosis and patient management. A tailored preconception carrier screening for our population would therefore need to be carefully constructed depending on what variants are present and the nature of the associated genetic diseases. Interestingly, all the significantly enriched variants in Acadians are currently covered by most commercial carrier screening panels, with the exception of *BHCE*. Although perhaps less relevant for preconception carrier screening, an enrichment of *BHCE* in Acadians may be interesting to measure in the context of precision medicine when administering anaesthesia. Our results are consistent with a founder effect in the Acadian population leading to increased frequency of some disease-causing variants. This study further reveals potential Acadian founder variants. Follow-up studies should aim to estimate distant kinship relationships based on genetic data. In addition, studies of additional regional populations are warranted to better elucidate the frequency of pathogenic variants and overall genetic architecture of Acadians throughout the Atlantic Canadian Provinces. This may lead to a better comprehension of variant frequency for developing a screening program aimed at preventing heritable disease in this region of Canada.

## Supplementary Information

**Additional file 1**. **Table S1**. Genes included in sequencing panel. **Table S2**. Cohort characteristics. **Table S3**. Genetic diseases associated with genes in which pathogenic variants have been detected. **Table S4**. Allele frequency (AF) and heterozygotes frequencies (HF) comparisons with higher risk populations on gnomAD. **Table S5**. Hardy-Weinberg comparison.

**Additional file 2**. **Figure S1**. Probability of detecting at least one variant as a function of allele frequency for a sample of 60 individuals. The minor allele frequencies in the reference population for the variants observed in our sample range from 9 × 10-6to 0.5.

## Data Availability

Our study population represents a vulnerable group, and may be at greater risk of genetic discrimination, stigmatisation, and breach of privacy. Genomic data has therefore been restricted due to higher risk of re-identification from genetic information.
