# Supplementary material for: Pathogenic variants carrier screening in New Brunswick: Acadians reveal high carrier frequency for multiple genetic disorders
Source: BMC Med Genomics. 2022 Apr 29;15:98. doi: 10.1186/s12920-022-01249-1 (PMC9055701; doi:10.1186/s12920-022-01249-1)
Supplement: Supplementary file 2 — Additional file 2. Figure S1. Probability of detecting at least one variant as a function of allele frequency for a sample of 60 individuals. The minor allele frequencies in the reference population for the variants observed in our sample range from 9 × 10^-6^to 0.5. [file 12920_2022_1249_MOESM2_ESM.pdf]

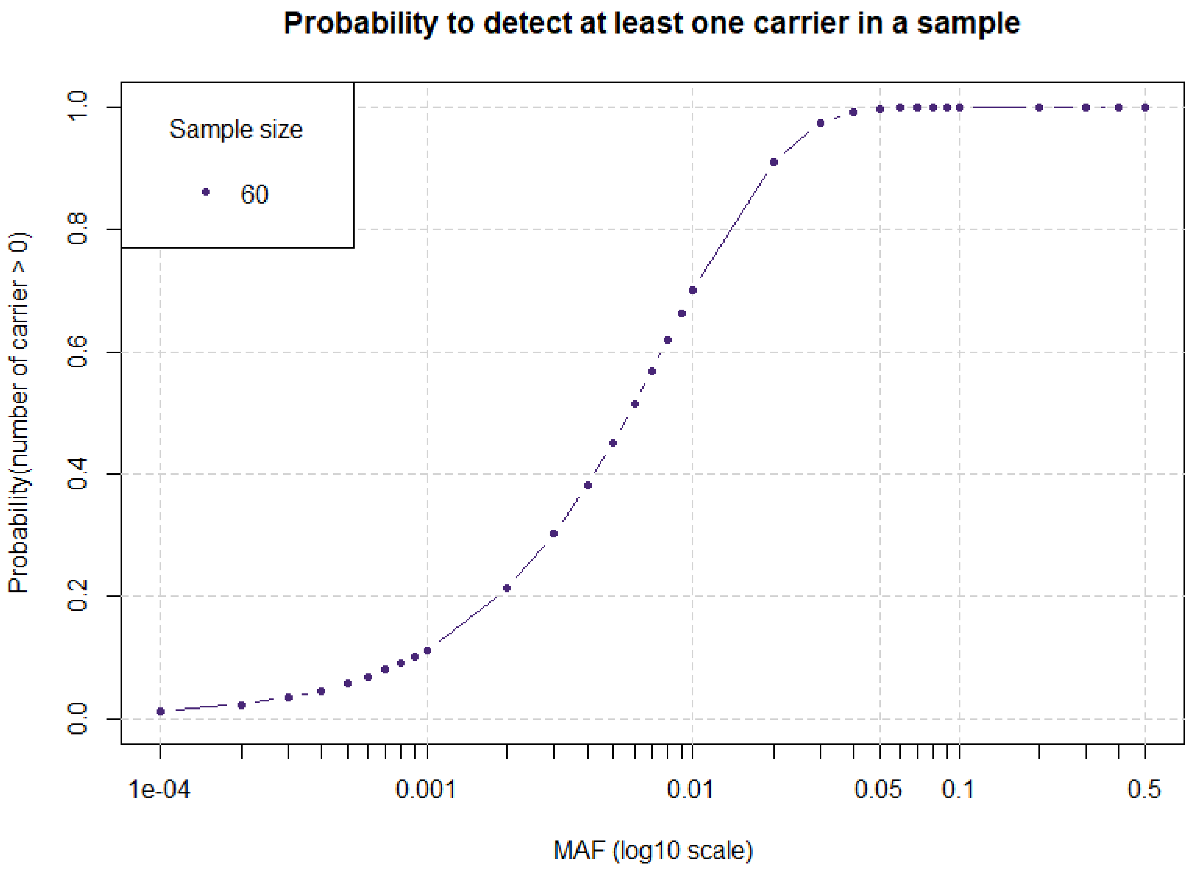

Figure S1: Probability of detecting at least one variant as a function of allele frequency for a sample of 60 individuals. The minor allele frequencies in the reference population for the variants observed in our sample range from 9×10^{-6} to 0.5.
